# To Prevent Lockjaw

**Published:** 1912-09

**Authors:** Chas. T. Souther


TO PREVENT LOCKJAW.—Recently several articles have emphasized the value of magnesium sulphate in the treatment of tetanus. Another author reports thirty deaths, gathered from literature, after the administration of $MgSo_4$ by mouth and goes on to analyze these cases and ascribes death to a motor paralysis due to over absorption of $MgSo_4$ in cases where it fails to produce any catharsis. All the deaths were in cases where no catharsis resulted. On this heretofore little emphasized physiological action of $MgSo_4$ is based its action in tetanus. Horace Greeley, M. D., of Brooklyn, reported the cure of two well-marked cases of tetanus by the injection under the skin of $MgSo_4$, drahms ii; water dist., O i. Use same as normal salt solution. A very decided action was noted in a half hour. Injections can be repeated every six hours and the opisthotonos disappears and can be controlled by repeated injections. It will be remembered in this connection that much work has been done with $MgSo_4$ as a spinal cord anesthetic. The value of .aspirating the spinal fluid in those cases where there is any evidence of coma or increased cerebral pressure should not be overlooked. Dr. Benjamin, Camden, N. J., (*Medical Record*) claims to be able to always prevent the development of tetanus. He has not had a case develop in 10,000 injuries, including rusty nails and all kinds of punctured wounds. His treatment in substance is to sterilize the field of injury, irrigate large wounds, cocainize with an alcoholic solution of cocaine. Have on hand a set of screws (all sizes of ordinary screws will do); (1) dip the screw into 2 to 3 per cent lysol solution and screw into the wound, but hold back so as to turn several times, but not exceed the depth of the injury; (2) then remove the screw and use a fresh screw and dip in tincture of iodine and introduce to the bottom of the wound and turn backward a few times; (3) then dip screw in 7 per cent (1 to 14 parts) of carbolic acid in olive oil and make the third application in the same way; (4) sterile dressing and rest.—*Chas. T. Souther, M. D., in Lancet Clinic.*

